# Marine Natural Products, Multitarget Therapy and Repurposed Agents in Alzheimer’s Disease

**DOI:** 10.3390/ph13090242

**Published:** 2020-09-11

**Authors:** Márcia Martins, Renata Silva, Madalena M. M. Pinto, Emília Sousa

**Affiliations:** 1 CIIMAR—Centro Interdisciplinar de Investigação Marinha e Ambiental, Terminal de Cruzeiros do Porto de Leixões, 4450-208 Matosinhos, Portugal; up201605865@ff.up.pt (M.M.); madalena@ff.up.pt (M.M.M.P.); 2 Laboratório de Química Orgânica e Farmacêutica, Faculdade de Farmácia, Universidade do Porto, Rua de Jorge Viterbo Ferreira 228, 4050-313 Porto, Portugal; 3 UCIBIO-REQUIMTE, Laboratório de Toxicologia, Faculdade de Farmácia, Universidade do Porto, Rua de Jorge Viterbo Ferreira 228, 4050-313 Porto, Portugal; rsilva@ff.up.pt

**Keywords:** Alzheimer’s disease, marine natural products, drug combination, multitarget-directed ligand therapy, drug repurposing

## Abstract

Alzheimer’s disease (AD) is a multifactorial disease characterized by the presence of amyloid plaques, neurofibrillary tangles, and nerve cell death that affects, mainly, older people. After decades of investigation, the search for an efficacious treatment for AD remains and several strategies can be and are being employed in this journey. In this review, four of the most promising strategies, alongside with its most promising agents under investigation or development are highlighted. Marine natural products (MNP) are a source of unique chemical structures with useful biological activities for AD treatment. One of the most promising compounds, a marine-derived acidic oligosaccharide (GV-971) just passed phase III clinical trials with a unique mechanism of action. Combination therapy and multitargeted-directed ligand therapy (MTDL) are also two important strategies, with several examples in clinical trials, based on the belief that the best approach for AD is a therapy capable of modulating multiple target pathways. Drug repurposing, a strategy that requires a smaller investment and is less time consuming, is emerging as a strong contender with a variety of pharmacological agents resurfacing in an attempt to identify a therapeutic candidate capable of modifying the course of this disease.

## 1. Alzheimer’s Disease

### 1.1. Facts and Characteristics

Alzheimer’s disease (AD) is considered, by the World Health Organization (WHO), a global health priority [1]. By the year 2050, a new AD case is expected to be diagnosed every 33 s, with a total of approximately one million new cases per year [2]. AD is a neurodegenerative disease and is thought to commence years before the appearance of symptoms, which include memory loss and language problems. The origin of the symptoms is associated with the destruction or damage of neurons involved in cognitive function. With the progression of AD, some functions, such as walking and swallowing, become impossible and, in the end, people need around-the-clock care [3]. Recognized risk factors for AD are family history, advanced age, the apolipoprotein E (APOE) ε4 allele genotype, illiteracy, cardiovascular disease risk factors, lifestyle, and psychosocial factors [3].

AD is characterized by the presence of extracellular amyloid plaques, intracellular neurofibrillary tangles (NFT), and nerve cell death, which leads to brain atrophy [3,4]. β-Amyloid (Aβ) plaques may contribute to cell death by interfering with the communication at synapses between neurons, while NFTs block the transport of nutrients and other essential molecules within neurons [3].

**Figure 1:**
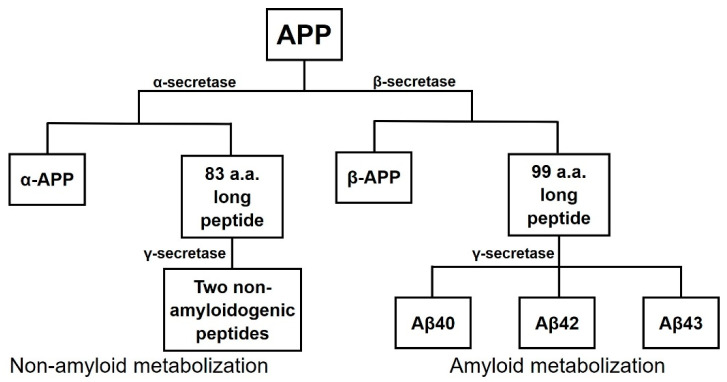
Scheme of cleavage of amyloid precursor protein (APP) through two possible metabolic pathways, the non-amyloid metabolization and the amyloid metabolization. Adapted from [5].

The amyloid plaques are mainly composed of Aβ peptides resulting from the cleavage of the amyloid precursor protein (APP). APP can be cleaved by two distinct metabolic pathways, the non-amyloiddogenic and the amyloidogenic pathways, by the action of three secretases (Figure 1). In the non-amyloidogenic pathway, the involved enzymes are α-secretase and γ-secretase. First, APP is cleaved by α-secretase that generates a soluble fragment named α-APP and a smaller 83 amino-acid (a.a.) long peptide that is then cleaved by γ-secretase into two non-amyloidogenic peptides. The second pathway is the one that generates Aβ peptides since the cleavage occurs at the N-terminus of the Aβ peptide of the fragment. β-secretase (BACE-1) cleaves APP producing two fragments: β-APP, a soluble fragment, and a longer 99 a.a. peptide that is then cleaved by γ-secretase into amylogenic peptides of varying length, including Aβ40, Aβ42, and Aβ43 [5].

NFTs are formed from hyperphosphorylated tau protein, a protein that stabilizes the microtubules but which, when hyperphosphorylated, accumulates into tangles [4,6]. The main enzyme involved in the process is glycogen synthase kinase 3β (GSK-3β) and its inhibition can lead to a reduction in tau hyperphosphorylation. GSK-3β can be inhibited by protein kinase C (PKC), which is stimulated by muscarinic receptor 1 (M1) agonists such as acetylcholine [5].

**Figure 2:**
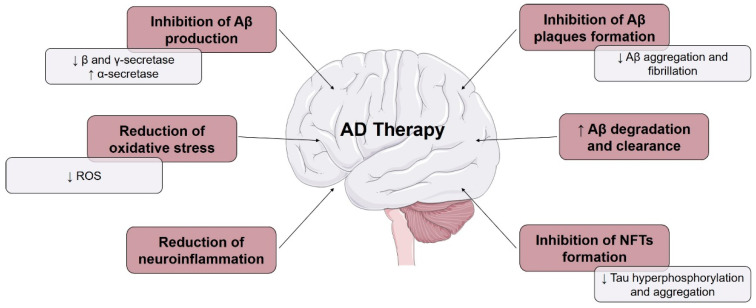
Several strategies used in Alzheimer’s disease (AD) therapy. Arrows pointing up and down indicate increase and decrease, respectively. Adapted from [10].

The presence of toxic Aβ peptides and tau proteins causes an immune system response that activate microglia. These cells try to eliminate the toxic compounds, and when the production is higher than the clearance, inflammation occurs [3]. An abnormal increase in the production of reactive oxygen species (ROS) leads to oxidative stress, which is another major contributor to the development of AD. The presence of certain metal ions, such as aluminum (Al^3+^), iron (Fe^2+^), copper (Cu^2+^), zinc (Zn^2+^), and mercury (Hg^2+^), and mitochondrial dysfunction, also contribute to the pathogenesis of AD [7]. Recently, studies in mice investigating the possibility that Aβ peptide from the intestinal microbiome might be a trigger leading to AD opened a new avenue of research in this area [8,9]. Therefore, AD can be caused by several mechanisms, leading to the possibility of being treated by different pathways, which are outlined in Figure 2 [10].

### 1.2. Approved Therapies

The first AD case was reported in 1907 by Alois Alzheimer [1] and, despite the impact on society, the current therapies only relieve symptoms, with no disease-modifying therapies or treatments (DMT’s) currently existing [4,6].

**Figure 3:**
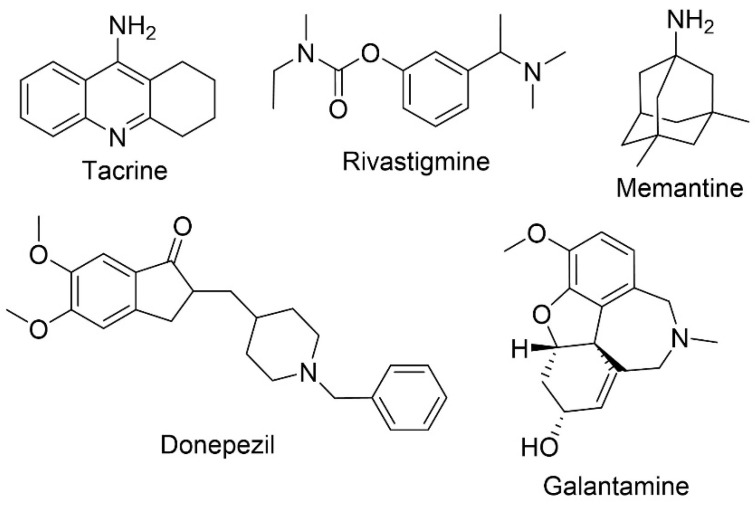
Chemical structures of the approved therapies for AD, tacrine, rivastigmine, memantine, donepezil, and galantamine.

Five drugs have been approved for the treatment of AD (Figure 3). Four of these drugs are acetylcholinesterase inhibitors (AChEI) namely, tacrine (approved in 1993 and discontinued due to adverse effects), donepezil (in therapy since 1996), rivastigmine (approved in 1998) and galantamine (approved in 2001). The fifth drug is a *N*-methyl-d-aspartate (NMDA) receptor antagonist, memantine (approved in 2004) [11,12]. To modulate the behavioral symptoms, antipsychotic and antidepressant treatments are also administrated [13].

Cholinergic systems are affected early in the disease process, resulting in a decrease of acetylcholine in neurons. This also interferes with the function of enzymes involved in the synthesis and degradation of acetylcholine, a neurotransmitter important for nerve cells communication [13]. The objective of AChEIs is, therefore, to increase the levels of acetylcholine through the blockage of acetylcholinesterase (AChE), the enzyme responsible for its degradation [13,14]. Studies have revealed that the three AChEIs used in clinical practice (donepezil, rivastigmine, and galantamine) are equally efficacious to treat mild to moderate AD [15]. However, the use of this class of drugs is associated with several adverse effects, such as increased rate of syncope and bradycardia. Donepezil, when compared to rivastigmine and galantamine, presents less adverse gastrointestinal adverse effects [13].

Both amyloid plaques and NFT can possibly cause the overstimulation of glutamate, a primary excitatory a.a. of the central nervous system (CNS), leading to a tonic activation of the NMDA receptor that in turn would result in excitotoxicity and neuronal degeneration. Memantine, a NMDA receptor antagonist, reduces calcium influx into the cell through the modulation of glutamate activity [16]. The most common adverse effects are dizziness, headache, and confusion, although a small percentage of patients can also develop agitation [13]. AChEIs and memantine mechanisms of action are different, and dual therapy is often prescribed for the management of AD. Normally, memantine is subsequently added to the ongoing treatment with AChEIs that generally commenced months or years prior. Combination therapy studies demonstrated an improvement in cognitive functions, behavioral symptoms, and prolonged functional independence in patients with moderate to severe AD, when compared to the monotherapy [17,18].

Although the discovery of new therapies for AD is crucial, the results found so far do not correspond with the expected success; it should be noted that the causes related to this situation cannot be associated with the lack of knowledge in terms of pathophysiology and biology of CNS disorders [19].

## 2. Current Approaches and Objectives

As mentioned, Aβ peptide is formed by the action of β- and γ-secretase and when production increases or the clearance decreases, Aβ peptide accumulates in CNS leading to oligomerization, and to the formation of Aβ plaques causing synaptic dysfunction and neurodegeneration [12,20,21]. Some molecules can inhibit the aggregation of the Aβ peptide or bind to Aβ monomers maintaining them in a non-fibrillar form. α-secretase may be a potential therapeutic target, because its increase promotes the non-amyloidogenic pathway, reducing the formation of amyloidogenic Aβ peptides. In addition, it also promotes the formation of α-APP, a potential neuroprotective protein. BACE-1 activity is overexpressed in AD, increasing the formation of amyloidogenic peptides and, consequently, neurodegeneration. The inhibition of BACE-1 might reduce this process. In addition to catalyzing the second step of the amyloidogenic pathway, γ-secretase is also responsible for the formation of Notch protein, which is important for cell development and survival. As a result of this important function, the objective is to modulate the activity of the enzyme instead of causing its inhibition [20]. The formation of Aβ plaques causes neuroinflammation, oxidative stress, and alters kinase and phosphatase activity, leading to tau hyperphosphorylation and the formation of NFT [12]. The inhibition of GSK3β might be also a possible target [20]. Other potential targets are butyrylcholinesterase (BChE) and monoamine oxidase (MAO) A and B enzymes [13]. BChE is present in high concentrations in AD patients and can be considered responsible for the degradation of acetylcholine. MAO A is responsible for the deamination of certain neurotransmitters, such as serotonin, noradrenaline, and dopamine. MAO B causes degradation, mainly, of phenyl ethylamine and benzylamine. Both isoforms are associated with AD pathology and are possible targets. MAO A is associated with toxicity and neuronal death and MAO B is associated with Aβ peptide [22]. Other processes, such as oxidative stress, neuroinflammation, calcium and metal dyshomeostasis, excitotoxicity, and mitochondrial damage, are also considered important for drug development [13].

According to Cummings et al. [23], in 2019, there were 132 agents in 156 trials of anti-AD therapies: 28 agents in phase III, 74 agents in phase II, and 30 in phase I. Of all the clinical trials, 19 agents targeted cognitive enhancement, 14 aim to treat neuropsychiatric and behavioral symptoms and 96, the majority, intended to become a DMT and had amyloid as their primary target [23].

**Figure 4:**
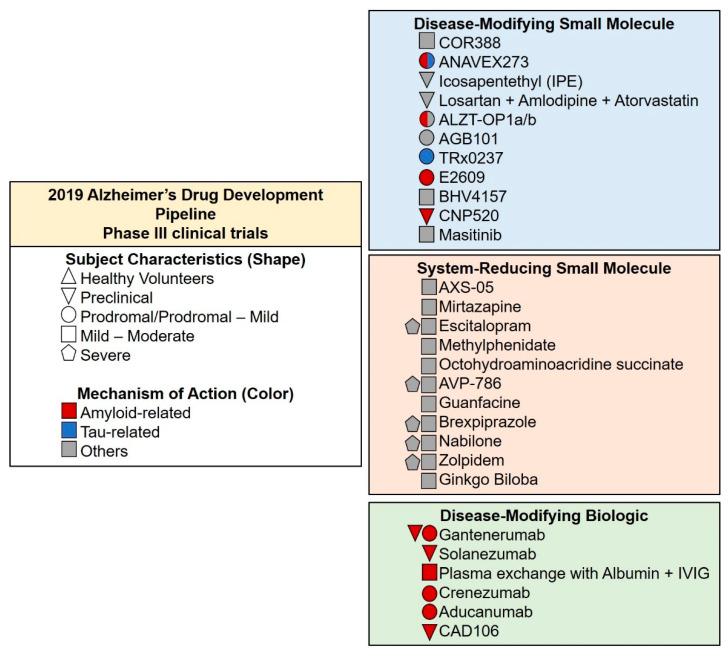
Compounds in phase III clinical trials in 2019, classified by mechanism of action. The agents in green are biologic, in blue are small molecules, and in orange are symptomatic agents designed to treat AD associated symptoms or cognitive enhancement. Adapted from [23].

There are currently 28 agents being tested in 42 phase III clinical trials (Figure 4). Eight agents target neuropsychiatric symptoms such as agitation, apathy, and sleep disorders (AVP-786, AXS-05, brexpiprazole, escitalopram, methylphenidate, mirtazapine, nabilone, and zolpidem) and three agents are being studied as symptomatic cognitive enhancers (*Ginkgo biloba*, guanfacine, octohydroaminoacridine succinate). Relating to DMT, three possess neuroprotection activity (ACB101, BHV4157, ethyl eicosapentaenoic acid) and three anti-inflammatory properties (COR388, losartan+amlodipine+atorvastatin, and masitinib). Nine are anti-amyloid agents (gantenerumab, cad106, aducanumab, solanezumab, plasma exchange with albumin+IVIG, crenezumab, E2609, ALZT-OP1a/b, and CNP520), one anti-Tau (TRx0237), and one possesses both anti-amyloid and anti-Tau activity (ANAVEX2-73) [23].

Over the last few years, drug discovery in AD has focused on DMT, treatments with the objective of modifying the disease process by impacting characteristic brain changes. Numerous candidates failed in clinical trial development and no DMTs showed a significant difference in phase III clinical trials or received marketing approval [24]. The most studied hypothesis is the amyloid hypothesis, but the lack of successin identifying targeted therapeutics has led to the abandoning this idea and a focus on other therapies, such as combination therapy [23]. In AD, a single-target high affinity drug approach is unlikely to be effective. As AD is a neurodegenerative disease caused by various pathways, it is currently believed that the best approach is to identify a drug capable of modulating multiple target pathways [25]. The most promising compounds for this strategy are nature-derivatives, because they can interact with a wide range of molecular targets [26] and present a higher resemblance to biosynthetic intermediates or endogenous metabolites than do synthetic compounds [27].

In this review, strategies to identify a promising treatment for AD will be highlighted. From the various sources of natural products (NP), marine natural products (MNPs) have excelled in clinical trials. The focus therefore will be mainly on MNPs that have presented the ability to improve AD symptoms, since it is also an unexplored environment full of new interesting secondary metabolites. Alongside the most promising MNPs that are in the pipeline for AD treatment, two other emergent approaches in AD drug development will be highlighted, namely polypharmacology and drug repurposing. Examples of agents from these strategies with promising activity that are being currently studied will also be identified

## 3. Marine Natural Products

Secondary metabolites of terrestrial and marine organisms are important sources for the discovery of new drugs. In the last 20 years, approximately 50% of the drugs introduced into the market were directly derived from small molecules with natural origin [28]. Recent studies revealed that, among several biological activities of NP, neuroprotective activity is one very useful property that can act to prevent and attenuate neurological diseases [10]. The majority of drugs from natural organisms are obtained from terrestrial sources [28]. However, marine organisms can be considered, from an industrial point of view, the most recent source of bioactive compounds. Oceans are a unique and rich source of useful active compounds, especially for the pharmaceutical industry, due to the association of a large genetic diversity of organisms and a huge ecological and molecular diversity. To survive conditions of specific physical and chemical properties such as pressure, temperature, salt concentration, light penetration, radiation exposure, and oxygen concentration, marine organisms are in constant adaptation. As a result of that, they can synthesize different metabolites, starting from small molecules to more complex structures as peptides and enzymes, usually used to immobilize and capture predators [29].

The problems associated with marine compounds are their complex chemical structures, which are difficult to synthesize, and their limited quantities from original sources [30]. The exploration of marine environment is increasing since new techniques for sample collection have been developed, and spectrometric techniques and separation methods have improved [31].

Despite their limitations, marine compounds are being increasingly explored, leading to the emergence of new chemical structures with promising therapeutic characteristics such as those with anti-inflammatory, analgesic, immune-modulatory, neuroprotective, and anticancer effects, among others [28,32].

In the following sections we discuss some MNPs that are either in clinical trials or have potential for AD treatment.

### 3.1. Homotaurine

Proteoglycans are involved in Aβ aggregation and amyloid fibril formation. The sulfated glycosaminoglycan chains, components of proteoglycans, bind to Aβ peptide contributing to the transition of Aβ from random-coil to a β-sheet conformation, contributing to the process of fibrillogenesis [33]. A screening of low molecular weight sulfated molecules was developed to mimic the ionic properties necessary for the binding of glycosaminoglycans to Aβ. Homotaurine (Table 1, entry 1), a small amino sulfonate (3-amino-1-propanesulfonic acid) identified in marine red algae, was discovered, and was shown to inhibit Aβ aggregation and fibrillogenesis in vitro [33,34]. This glycosaminoglycan mimetic interacts with Aβ monomers and maintains them in a stable conformation, preventing the progression of the amyloid cascade [35]. Studies have suggested that the binding of homotaurine to Aβ peptide occurs in the sulfonated head group of the molecule, instead of the amino acid end of the compound [35].

**Figure 5:**
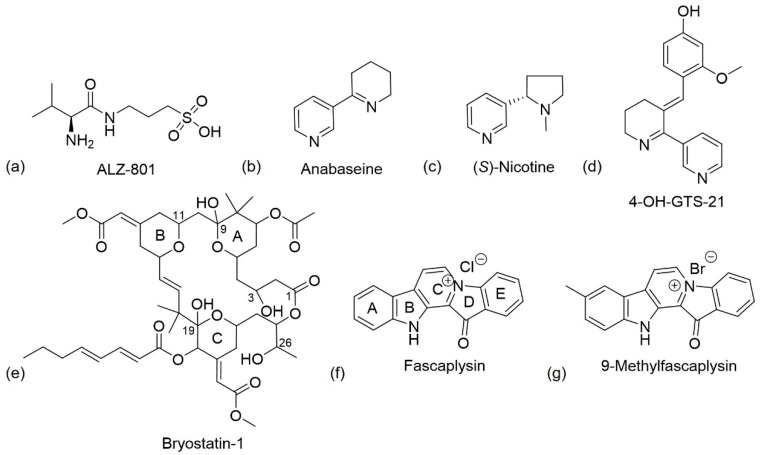
(**a**–**d**,**g**) Chemical structure of ALZ-801, (*S*)-nicotine, anabaseine, 4-OH-GTS-21, and 9-methylfascaplysin, respectively. (**e**) Chemical structure of bryostatin-1. Structure-activity relationship (SAR) studies indicated that the presence of the 20 membered macrolactone ring, C-26 free hydroxyl, and C-1 carbonyl are essential, and C-3 is important for molecular conformation. Alterations to form analogues can occur at C-20 and to alter pharmacokinetics, it is possible to change C-9. Adapted from [54]. (**f**) Chemical structure of fascaplysin. According to SARs studies, the quaternary status of the C-ring nitrogen is essential for P-gp activity [59].

Homotaurine has been evaluated in phase III clinical trials to treat mild to moderate AD but the results were unsatisfactory due to its reduced clinical efficacy. Despite this failure, post hoc analyses revealed a reduced cognitive decline and reduced hippocampal volume loss in treaded patients, that was more prominent in a homogeneous population of APOE4 patients [36]. The oral administration of this drug, although promising improvements to susceptible AD populations, possess high interindividual pharmacokinetic variability and causes nausea and vomiting, probably due to direct gastrointestinal irritation. To overcome these adverse effects, a valine-conjugated prodrug of homotaurine, ALZ-801 ((*S*)-3-(2-amino-3-methylbutanamido)propane-1-sulfonic acid) was developed (Figure 5a). This drug is rapidly absorbed in the gastrointestinal tract and the valine moiety is cleaved via amidases, thus releasing homotaurine. The prodrug had a longer half-life and smaller pharmacokinetic variability than homotaurine [66]. A phase III clinical trial for ALZ-801 in individuals homozygous for APOE4 is planned [67].

### 3.2. Anabaseine

Nicotinic acetylcholine receptors (nAChRs) are pentameric membrane proteins that belong to a family of ligand-gated ion channel receptors. They are composed of different combinations of five subunits (α, β, γ, δ and ε) that form cysteine-loop ligand-gated cation channels permeable to Na^+^ and K^+^ and, to variable amounts of Ca^2+^. In the brain, the most abundant subunits are homomeric α7 subtype and the heteromeric α4β2 subtype, which are highly expressed in brain regions that develop AD neuropathology [68,69,70]. The binding of an agonist to a α7 nAChR leads to conformational changes and, consequently, the opening of the channel allowing the passage of ions. Following activation, the channel rearranges resulting in a loss of biological response [71]. The nAChR is a target for AD, because Aβ binds to these receptors, especially to the α7 subtype, and can alter the normal functionalization of the receptor leading to the initialization of Aβ [68].

Anabaseine (Figure 5b), a natural nicotine-related pyridine alkaloid, was the first nemertine alkaloid to be isolated and identified [72]. It was initially isolated from a marine worm, but it is also found in certain ants. The chemical structure is similar to nicotine (Figure 5c), but it contains an imine double bond, instead of a saturated piperidine ring. It is a non-specific nicotinic agonist that, like nicotine, stimulates all nAChRs with different affinities, with anabaseine presenting a higher affinity for α7 nAChRs [38]. The addition of a benzylidene substituent to the 3-position of the anabaseine tetrahydropyridine ring creates a benzylidene-substituted anabaseine with functional selectivity for α7 nAChRs [73].

Some studies used computational methods, 2D- and 3D- quantitative structure–activity relationship (QSAR) and molecular docking to determine how the substituents on the arylidene ring affect the interaction of anabaseine with α7 nAChRs [69,73]. The ligand binding pocket is composed of five amino acids with electron-rich aromatic side chains, Tyr92, Trp147, Tyr188, Tyr195, and Tyr 55. A Cloop protects the binding pocket from the outside solvent, and it contains Cys189 and Cys190 residues in the tip linked by a disulfide bridge, a characteristic of nAChR α-subunits [73]. QSAR and docking studies predicted the importance of the nitrogen atom in the tetrahydropyridyl ring for hydrogen bond formation with Trp145, the π-π interaction between the aromatic ring of the ligand and the binding site, and the molecular surface recognition expressed in terms of steric complementarity. The 3D-QSAR indicated that bulky substitutions at positions 2 and 4 of the benzylidene moiety, with atoms with high electronegativity such as a methoxyl group, enhance binding affinity to the α7 receptor [69].

The 3-arylidene-anabaseines have been shown to possess both neuroprotective and cognitive enhancing properties [38]. Remarkable examples are GTS-21 or DMXBA, 3-(2,4-dimethoxybenzylidene)-anabaseine, (Table 1, entry 2) and 4-OH-GTS-21 (Figure 5d), 3-(2-methoxy,4-hydroxybenzylidene)-anabaseine, two synthetic analogues of anabaseine, that have demonstrated to be more potent and selective than the parent compound, anabaseine. The two rings of anabaseine are co-planar due to the electronic conjugation. In the case of DMXBA, the three rings are predicted to be positioned in different planes [38]. GTS-21 is a lipophilic compound with a high permeation through the BBB. However, it is metabolized in vivo to generate the hydroxylated metabolite 4-OH-GTS-21, formed by *O*-demethylation. These metabolites showed better efficacies than GTS-21 on α7 receptors in mammalian species but they possess poor penetration into the brain probably due to their high polarity. As a result of this, it appears that orally administered DMXBA might lead to stimulant activity [37].

Analogues of anabaseine showed neuroprotective effects against amyloid toxicity in rat (e.g., PC 12 cells [74]) and human cells, and also protection from apoptosis and necrosis in rat neurons [39]. An in vitro study also demonstrated that the long-term administration of GTS-21 reduced the amount of Aβ and improved cognitive functions in a transgenic mouse model of AD [40]. The activation caused by GTS-21 led to the inhibition of neuronal γ-secretase activity and promoted microglial Aβ phagocytosis [40]. GTS-21 is currently in phase II clinical trials [39].

### 3.3. DHA

The polyunsaturated docosahexaenoic acid (DHA) (Table 1, entry 3), an essential omega-3 fatty acid, is predominantly present in synaptic junctions in the CNS, and is also present in other cellular membranes [41]. It is essential for brain health and neurodevelopment, especially during prenatal brain development [43], and it can be obtained endogenously from the bioconversion of α-linolenic acid [75], or exogenously, from dietary products, such as fish [76]. Older individuals, especially AD patients, possess lower DHA levels in the brain, which can be associated with a deterioration of cognitive function [49]. The reduction of DHA levels can occur as a result of lipid peroxidation through free radicals, decreased dietary intake, or deficiencies in DHA biosynthesis by the liver [46]. DHA consumption is associated with a lower risk of developing AD [41,49].

Animal studies have reported that DHA reduces Aβ deposition, phosphorylation of tau, and neuritic pathology [44,45,48]. Some studies have suggested that Aβ reduction occurs because DHA shifts the cleavage of APP to the non-amyloidogenic pathway, decreasing γ-secretase and BACE-1 activity [43,51]. The therapeutic effects of DHA supplementation are dependent on the stage of AD. Administration of DHA to patients with mild to moderate AD did not change the rate of cognitive decline [41,47]. Nevertheless, positive effects were verified in patients in the early stage of AD [47]. Currently, DHA is in phase II clinical trials.

**Figure 6:**
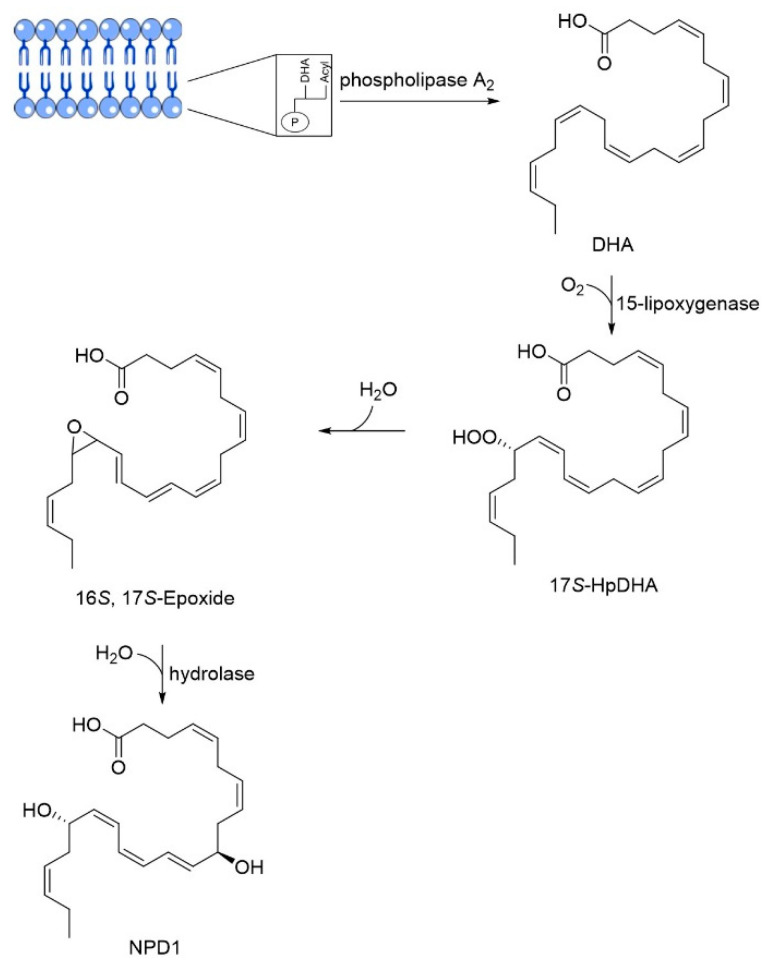
Biosynthesis of neuroprotectin D1 (NPD1). Phospholipase A2 releases docosahexaenoic acid (DHA) from membrane phospholipids and 15-lipoxygenase catalyzes the deoxygenation at C17, followed by the formation of an epoxide that is enzymatically hydrolyzed in NPD1. Adapted from [77].

Neuroprotectin D1 (NPD1), 10,17*S*-docosatriene, is a bioactive DHA-derivative formed by the conversion of DHA through the action of two enzymes, first by a phospholipase A2, and second by a 15-lipoxygenase-like enzyme (Figure 6) [46]. It has strong anti-inflammatory, anti-amyloidogenic and anti-apoptotic activities [46,50]. In vivo and in vitro, NPD1 suppressed Aβ42 formation from APP through the activation of α-secretase and downregulation of BACE-1, decreased inflammation through downregulation of pro-inflammatory genes and apoptosis, being beneficial for early stages of neurodegeneration [50].

### 3.4. Bryostatin

The first bryostatin, brysostatin-1 (Table 1, entry 4), was extracted in 1982 by Pettit et al. from *Bugula neritina* [78]. More analogues were then discovered during a search for new anticancer drugs. The structural elucidation of bryostatins was made by detailed spectroscopic and/or X-ray crystallographic analysis [79]. Bryostatins possess antitumor activity and immune modulatory properties, and also been demonstrated to be able to enhance memory and learning [56]. Bryostatin-1, a macrolide lactone with 11 chiral centers, is a potent modulator of PKC, a multifunctional protein responsible for cell differentiation and signal transduction [57]. Of the family of PKC isozymes, it was found that PKCε was involved in learning and memory and was deficient in fresh frozen hippocampal brain samples from AD patients [55]. Bryostatin-1 activates PKCε by binding to the *N*-terminus C1 domains of PKC causing autophosphorylation, protein translocation, and ubiquitination of PKC, thereby downregulating the protein. The normal levels are restored by de novo synthesis of the protein [52,57]. The activation of PKCε causes degradation of Aβ, activation of α-secretase, generating the synaptogenic non-toxic soluble amyloid-β protein precursor α and reduction of GSK3-β activity, thus decreasing hyperphosphorylation of tau [55]. Its neuroprotective effects may be related to its ability to induce synaptogenesis by increasing the levels of synaptic growth factors in the brain [52].

Structure-activity relationship (SAR) studies indicated that, for favourable PKC binding, the 20-membered macrolactone ring is essential, and the elimination of the A-ring and B-ring exocyclic olefin can occur. The C-26 free hydroxyl and C-1 carbonyl group are necessary for good interaction and affinity, respectively. The C-19 hydroxyl group may be involved in the interaction with the lipid bilayer and C-3 hydroxyl group is important for the molecule’s conformation. The C-9 region can be modified to alter pharmacokinetic characteristics, and C-20, a non-pharmacophoric site, can be used to form analogues (Figure 5e) [54].

A phase IIa safety and tolerability test showed that bryostatin-1 increases PKCε, is safe, has a favorable pharmacokinetic profile, and results in cognitive improvement with a single dose [52]. It is now undergoing phase II clinical trials for the treatment of AD.

### 3.5. Fascaplysin

Fascaplysin (Table 1, entry 5) is a fused benzoyl-linked β-carbolinium alkaloid isolated in 1988 from marine sponge *Fascaplysinopsis bergquist* sp. near the Fiji Island [58,59]. This marine compound demonstrated anticancer and antioxidative stress properties [80], but also anti-cholinesterase activity and induction of P-gp expression and activity [59]. P-gp, an important member of the ATP-binding *cassette* transporter superfamily, is related with AD once is an efflux transporter associated with Aβ transport out of the brain [81], and there is evidence that increased levels of P-gp result in and increased amyloid-β clearance [59]. These properties suggest that fascaplysin should be investigated as a therapeutic for AD.

Docking studies revealed that fascaplysin, a planar structure, binds to AChE active site, which possesses two sites of ligand binding, an acylation site at the end of the gorge and a peripheral site close to the entrance of the gorge. Fascaplysin binds in a hydrophobic pocket formed by Phe330, Tyr334, and Tyr121 residues and peripheral site residue Trp279. The principal interaction of fascaplysin with AChE is a π-π interaction between the E ring of fascaplysin and the residue Trp279 of the peripheral site. Fascaplysin binds parallelly in the AChE active site gorge, with its B-ring orientating towards the catalytic site and the D-ring orientating towards the peripheral site (Figure 5f) [58].

The P-gp induction activity of fascaplysin was discovered during an in vitro screening for P-gp modulation in a repository of natural products. Fascaplysin increases P-gp activity leading to an increase of Aβ efflux. SAR studies have already been performed. All analogues with quaternary nitrogen possessed activity even with the opening of the D-ring. The derivatives with an open D-ring without the quaternary nitrogen and the non-polar and non-quaternary derivatives were inactive. This indicates that the quaternary status of the C-ring nitrogen is essential for P-gp activity (Figure 5f) [59].

Based on molecular modeling studies, 9-methylfascaplysin (Figure 5g), a fascaplysin derivative, was synthesized [60,61]. Docking analyses suggested that the binding to AChE active site differs from fascaplysin, which only binds to the peripheral anionic site. The referred derivative possibly binds also to the catalytic site residue leading to an enhanced AChE inhibitory activity [60].

This derivative, with greater potential for inhibiting Aβ fibrilization than fascaplysin, inhibited Aβ peptide formation through direct inhibition and prevented the associated toxicity and subsequent neuronal death at nanomolar concentrations. 9-Methylfascaplysin and Aβ possibly interact through hydrogen bonding and hydrophobic interactions, including π-π interactions. It is thought that the direct interaction between this fascaplysin derivative and Aβ causes competition between the intramolecular bonds of Aβ aggregates leading to the inhibition of Aβ self-assembly [61]. Studies performed in vitro also showed its potential to activate antioxidant enzymes and thus, to produce neuroprotective effects against ROS. 9-Methylfascaplysin crossed the BBB in mice, preventing cognitive damages through the inhibition of AChE in the hippocampus, without causing severe neurotoxicity [60].

Studies in vitro and in vivo suggested that this molecule can prevent cognitive dysfunction, decrease neuroinflammation, and reduce tau hyperphosphorylation, but further investigations regarding its safety profile are required [60]. Fascaplysin derivatives can become a new class of potential multi-target drugs for AD.

### 3.6. GV-971

On 3 November 2019, Shanghai Green Valley Pharmaceuticals announced that China’s National Medical Product Administration had conditionally approved GV-971 (Table 1, entry 6) for the treatment of mild to moderate AD following successful completion of a phase III clinical trial. Full approval is expected in 2020 [62]. GV-971 is an orally administered mixture of acidic linear oligosaccharides, a marine-derived acidic oligosaccharide extracted from brown algae [63,65].

**Figure 7:**
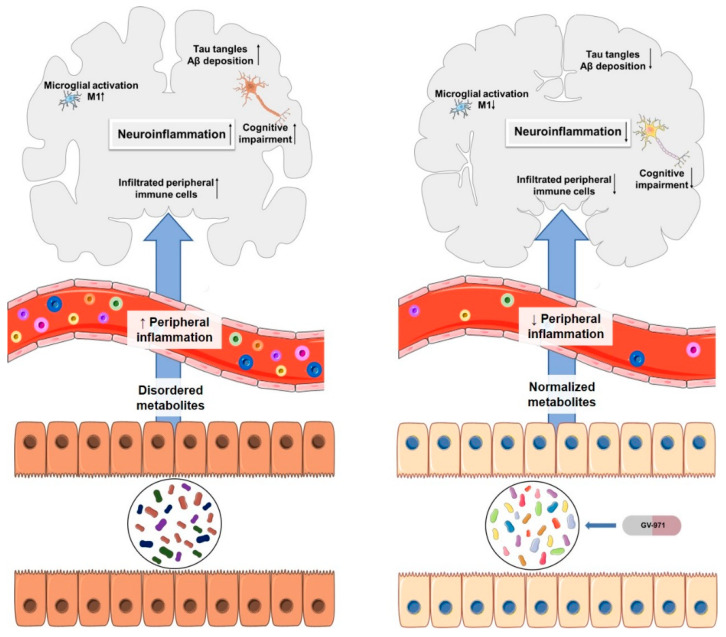
Association of AD and gut microbiota on the **left**. On the **right**, the effects of oral administration of GV-971. Adapted from [64].

This oligomannate is based on a new therapeutic strategy. The gut microbiota is involved in microglia activation and neuroinflammation in AD. Studies have reported that gut dysbiosis can alter the host immune responses causing various inflammatory disorders such as the activation of microglia during AD development [64]. Wang, et al. [64] proposed a possible mechanism in which the altered gut microbiota composition leads to an abnormal elevation of amino acids, principally phenylalanine and isoleucine. These amino acids increase the passage of Th1 cells through BBB via blood circulation. In the brain, Th1 cells may locally interfere with M1 microglial cells, resulting in neuroinflammation and cognitive impairment. GV-971 restores the normal microbial profile, reduces the concentration of phenylalanine and isoleucine and, consequently, reduces neuroinflammation associated with Th-1 cells (Figure 7) [63,64]. In vivo, GV-971 reduced Aβ deposition, tau phosphorylation, and improved cognitive function, an effect that was observed in phase III clinical trial patients as well. It also reduced Th1 cells, alleviating neuroinflammation, and the data suggests that these effects occur through gut dysbiosis modulation, as per the proposed mechanism [64]. In phase II clinical trials, sodium oligomannate was associated with an increase of Aβ levels in cerebrospinal fluid, suggesting increased amyloid clearance [65].

## 4. Multitarget Therapy

Many drugs currently used in therapy were discovered based on the one-molecule, one-target paradigm, which results in a highly selective ligand to a certain target. However, there are some diseases with multiple pathogenic factors, such as neurodegenerative diseases, and using more than one pharmacological approach can be highly advantageous [82].

In the past 15 years, most clinical trials concerned monotherapy, but many therapeutic agents under development have failed. Due to the complexity of AD, one possible successful strategy can be targeting more than one biological target [4]. The multitarget therapy can be achieved by two approaches. The first is through the administration of a mixture of monotherapies which includes a drug cocktail, constituting a combination of drugs with a single active agent, respectively, and drug combination where one formula is formed by multiple active agents. The second approach is referred to as multitarget-directed ligands (MTDL) where only one active ingredient is administered [83].

Combination therapy is associated with a higher risk of drug-drug interactions than MTDL therapy. These interactions are normally caused by the induction or inhibition of enzymes involved in drug metabolism, which can lead to changes in drug concentrations and consequently, lack of drug efficacy or toxicity. As previously mentioned, AD mainly affects older individuals, who ingest several drugs, a fact that can markedly increase the probability of drug-drug interactions. An advantage of MTDL is the simplification of the therapeutic regimen in a manner that is more convenient for the patient, especially for AD patients given their memory deficits. Additionally, the prediction of pharmacokinetic and pharmacodynamic properties are normally easier with a single agent, which also simplifies the manufacturing and formulation. As a result, the MTDL strategy appears more advantageous than combination therapy [84].

Despite the advantages of MTDLs, there are currently no drugs being tested in clinical trials for AD. In the following sections, we discuss combination therapies that are being tested in clinical trials where important MTDL compounds will be highlighted.

### 4.1. Combination Therapy

In this section, combination therapies in phase III clinical trials for AD treatment will be discussed (Table 2) [23]. 

#### 4.1.1. ALZT-OPT1

**Figure 8:**
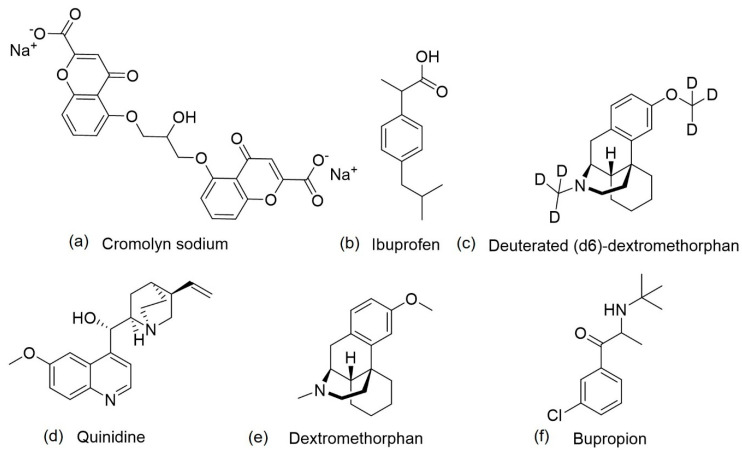
Chemical structures of agents that are being studied in phase III clinical trials, (**a**) cromolyn sodium, (**b**) ibuprofen, (**c**) deuterated (d6)-dextromethorphan, (**d**) quinidine, (**e**) dextromethorphan and (**f**) bupropion.

ALZT-OPT1 is a combination therapy that targets multiple disease pathways, namely amyloid and inflammation pathways, which is being tested in patients with early stage of AD. It is composed of two agents, cromolyn sodium (ALZT OP1a) (Figure 8a), an anti-amyloid agent, and ibuprofen, a non-steroidal anti-inflammatory drug (NSAID) (ALZT OP1b) (Figure 8b) [4]. Ibuprofen is administered orally, and cromolyn via dry powder inhaler [85].

Cromolyn sodium, a synthetic chromone derivative, is a mast cell stabilizer with anti-inflammatory activity that acts via the suppression of cytokine release, and has been approved by the FDA since the 1970s for asthma treatment [85,86]. In vitro studies demonstrated its ability to reduce Aβ fibrilization and oligomerization and to decrease soluble Aβ within the brain [86]. Cromolyn, alone or in combination with ibuprofen, converted the state of microglial activation form that which favors neuroinflammation to a state that promotes phagocytosis of Aβ peptides in AD transgenic mouse models [90]. Studies with ibuprofen reported its ability to reduce Aβ_42_ peptide levels by modulating γ-secretase activity, in addition to the inhibition of cyclooxygenase, the principal target of NSAIDs [89]. This explanation is attributed to the reduced prevalence of AD in individuals that use NSAIDs, as documented by epidemiological studies [89]. A clinical trial with ibuprofen in individuals with mild-moderate AD has already been performed. However, the results were not favourable since this drug did not slow cognitive decline [88]. As a result of the positive studies on cromolyn and ibuprofen, the idea of combining these two agents are now undergoing phase III clinical studies [23].

#### 4.1.2. AVP-786

Most AD patients suffer from agitation, a neuropsychiatric symptom that causes increased psychomotor activity, disruptive and/or aggressive behaviors, disinhibition, and emotional distress. This symptom is difficult to treat and causes an extreme reduction in the quality of life of both patients and caregivers. There are any drugs currently approved to treat agitation in AD [96], but some new candidates that are being investigated include AVP-786, which are in phase III clinical trials [23,91]. AVP-786 is a combination of deuterated (d6)-dextromethorphan hydrobromide (Figure 8c) and quinidine sulfate (Figure 8d). In 2013, the FDA granted AVP-786 a fast-track designation for the treatment of agitation in AD patients [91].

AVP-786 is a second version of AVP-923, also a combination therapy with the same active ingredients to treat pseudobulbar affect. The difference between the two therapies is related to the presence of deuterium, a heavier isotope of hydrogen in AVP-786 [91]. The active ingredients are dextromethorphan, or deuterated (d6)-dextromethorphan, and quinidine. Dextromethorphan (Figure 8e), a synthetic dextrorotatory methylated levorphanol analogue, was approved by FDA, in 1958 for its antitussive properties. It is a weak antagonist of the NMDA receptor, a sigma-1 receptor agonist, a serotonin and noradrenaline reuptake inhibitor, and a neuronal nicotinic α3β4 receptor antagonist [91,92,97]. Dextromethorphan is metabolized by cytochrome P450 2D6, reducing its effects in CNS. Therefore, to overcome this problem, it was formulated with another active ingredient, quinidine. Quinidine is an alkaloid stereoisomer of quinine, a NP extracted from the bark of the cinchona tree, with anti-arrhythmic properties that blocks voltage-gated sodium channels and inhibits cytochrome P450 2D6. This addition to the formulation increases dextromethorphan bioavailability through the reduction of first-pass liver metabolism and prolongation of plasma half-life, leading to increased neuropharmacological activity [91,92,98,99]. The results of phase II clinical trials of AVP-923 revealed that the compound was well tolerated and reduced the agitation in AD patients. However, the presence of quinidine is associated with drug-drug interactions and cardiac effects [91]. To overcome these effects, the most susceptible hydrogens of dextromethorphan (Figure 8e) were substituted with deuterium, increasing the strength of chemical bonds and consequently, decreasing the metabolism mediated by cytochrome P450 2D6, leading to a smaller required dose of quinidine, which reduces the adverse effects [94]. Use of the data generated with AVP-923 to support AVP-786 was permitted because of the similarity between the two compounds, as it had already been demonstrated that the pharmacological profile of deuterated (d6)-dextromethorphan and quinidine and dextromethorphan and quinidine were similar [91].

AVP-786 is also being studied for schizophrenia and in patients with traumatic brain injury, who suffer from behavioral disinhibition [91].

#### 4.1.3. AXS-05

AXS-05 is another compound that is in clinical trials for the treatment of agitation in AD patients. It is an oral, fixed-dose combination of dextromethorphan (Figure 8e) and bupropion (Figure 8f) with a similar mechanism of action to dextromethorphan/quinidine or deuterated (d6)-dextromethorphan/quinidine [93,94].

Dextromethorphan is rapidly metabolized by cytochrome P450 2D6 following oral administration. To increase its plasma levels, a combination of dextromethorphan and bupropion was established creating the agent AXS-05. Bupropion is a noradrenaline-dopamine reuptake inhibitor with an ability to inhibit cytochrome P450 2D6 and, consequently, in combination with dextromethorphan, increases its plasma levels [95]. Bupropion, approved for the treatment of depression in the United States in the late 1990s, is an aminoketone that inhibits the reuptake of both noradrenaline and dopamine, but without any effects on serotonin, histamine, acetylcholine, or adrenaline receptors [100].

The agents appear to have synergist effects [94]. Bupropion increases the amount of dopamine, and dextromethorphan increases serotonin and glutamate, given its antagonist NMDA receptor activity. Together, as AXS-05, they increase noradrenaline bioavailability [95]. This combination was found to be safe and well tolerated, resulting in a significant increase in dextromethorphan levels in plasma [93].

### 4.2. Multitarget-Directed Ligand Therapy

The focus on the strategy of “one molecule, multiple targets” is becoming one of the most interesting strategies for the development of an efficacious AD drug. The association between antioxidant and AChE inhibition activities is one of the most attractive approaches [101]. The most common strategy to generate MTDLs is based on the knowledge of the pharmacophore, where pharmacophores of ligands with affinities for different targets are combined into a single compound. The MTDLs can be classified into linked, fused and merged types. A linked MTDL constitutes pharmacophores connected through a linker that can be cleavable or non-cleavable. However, these MTDLs normally present with low bioavailability due to their large size. Fused MTDLs have partially overlapping pharmacophores while in merged MTDLs, the overlapping level is higher, which leads to a smaller molecular weight and simpler structures [83]. We will discuss examples of MTDLs for AD in the following section.

#### 4.2.1. Memoquin

**Figure 9:**
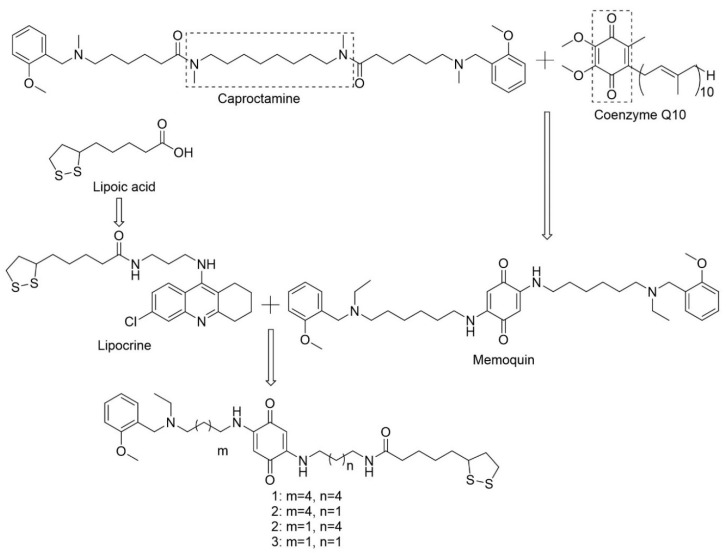
Design of memoquin through the conjugation of caproctamine and CoQ10, and design of lipoic acid memoquin derivatives through the conjugation of memoquin and lipocrine. Adapted from [104,105].

Memoquin, a 1,4-benzoquinone-poliamine hybrid compound, was one of the first rationally designed MTDL drug candidates for AD [84,102]. This MTDL was based on the polyamineamide caproctamine with anti-AChE and antimuscarinic activity, in addition to cholinergic properties. The authors added 1,4-benzoquinone, a synthetic derivative of coenzyme Q10, with potent mitochondrial antioxidant activity and neuroprotection against Aβ peptides in the hippocampus. The benzoquinone nucleus replaced the inner polymethylene chain resulting in the formation of memoquin (Figure 9) The hydrophobic and planar π system was able, in principle, of perturbing protein-protein interactions in the fibrillogenesis process of Aβ [103,104].

Docking studies confirmed the ability of memoquin to bind simultaneously to the catalytic site and the peripheral site of AChE. The residues Trp86 and Trp286 at the catalytic site and peripheral site of the enzyme make contact with the benzylammonium ends of memoquin. Some hydrogen interactions may occur between the quinone moiety and residues Tyr124 and Tyr341 [102]. In vitro, memoquin demonstrated the ability of inhibiting AChE, inhibiting AChE-induced Aβ aggregation, inhibiting BACE-1, and also presented antioxidant properties, reducing the formation of free radicals. The NAD(P)H:quinone oxidoreductase 1 (NQO1) enzyme catalyzes the two-electron reduction of 1,4-benzoquinone moiety to 1,4-dihydroquinone in vivo, increasing its antioxidant potential and scavenging properties [102,103,106]. NQO1 is increased in AD and, as memoquin is specifically reduced by this enzyme into the respective hydroquinone, it is possible that memoquin exerts its antioxidant activity specifically in brain regions affected by AD [103]. In in vivo studies, memoquin showed good oral bioavailability, ability to cross BBB, and presented a good safety profile, being well tolerated following prolonged administration [102]. It restored cholinergic deficit, reduced Aβ expression and accumulation, and reduced hyperphosphorylation of tau protein [102,103].

In 2009, the same investigation team synthesized new hybrid lipoic acid memoquin derivatives. α-Lipoic acid is a mitochondria-targeted antioxidant, which can help delay mitochondrial decay and, consequently, prevent or treat AD. The conjugation of α-lipoic acid and a 9-amino-6-chloro-1,2,3,4-tetrahydroacridine moiety led to the formation of lipocrine, an MTDL with antioxidant properties and AChE inhibitory activity. The conjugation of lipocrine and memoquin led to the development of four new MTDLs (Figure 9) with multiple antioxidant mechanisms and better potential for AD treatment and prevention. Studies performed in vitro and in vivo demonstrated that the derivatives present antioxidant activity reducing ROS production, but this activity was more pronounced following their reduction by NQO1. As memoquin and lipoic acid memoquin derivatives are composed of the same benzoquinone nucleus, it is possible that the antioxidant mechanisms of action are similar and mediated by NQO1. However, the derivatives also presented antioxidant activity in their oxidized form, probably due to the lipoic acid moiety that does not require activation of the enzyme to perform its antioxidant activity. The inhibition of AChE and BChE mediated by the derivatives was smaller when compared to memoquin, which suggests that the insertion of the lipoyl fragment in position two of the benzoquinone is prejudicial to the interaction with the enzymes [105].

#### 4.2.2. Dual BACE-1/GSK-3β Inhibitors

**Figure 10:**
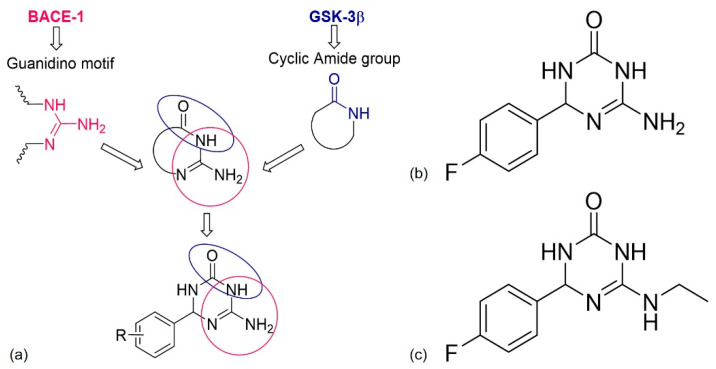
(**a**) Design strategy of dual β-secretase (BACE-1)/ glycogen synthase kinase 3 (GSK-3β) inhibitors that combines the pharmacophoric features for the BACE-1 and GSK-3β binding; (**b**,**c**) chemical structures of the most promising derivatives. Adapted from [107].

BACE-1 is the catalytic enzyme responsible for Aβ formation, and GSK-3β is responsible for tau hyperphosphorylation causing NFTs [107]. Both BACE-1 and GSK-3β are key targets for AD drug development and the first class of dual-target compounds that inhibit both enzymes, 6-amino-4-phenyl-3,4-dihydro-1,3,5-triazin-2(1*H*)-ones emerged through a ligand-based strategy [107,108]. The combination of the pharmacophoric features for BACE-1 (guanidino motif) and GSK-3β (cyclic amide group) binding into a single scaffold and docking study, generated the first class of dual BACE-1/GSK-3β inhibitors (Figure 10a) [107]. The guanidino motif must bind to the catalytic aspartic dyad of BACE-1, and the amide group with the amino and carbonyl functionalities must form hydrogen interactions with the GSK-3β binding site [109].

Some derivatives were synthesized and those with the highest inhibition activity contained the halogens fluor and bromo, probably because of the occurrence of polar and hydrophobic interactions with their targets. The chemical structure of the most promising compound is presented in Figure 10b [107]. It is a simple chemical structure, which increases the possibilities for functionalization and further chemical tractability [108]. In vitro, inhibitory activity was observed within the micromolar range for both enzymes, good brain exposure, neuroprotection, neurogenesis, and immunomodulation without toxicity [107]. Some modifications were performed in order to identify other promising derivatives and a very promising compound, 6-(ethylamino)-4-(4-fluorophenyl)-3,4-dihydro-1,3,5-triazin-2(1*H*)-one (Figure 10c), was discovered. It showed all the activities of the previous compound but with improved function [108,109].

#### 4.2.3. Xanthone and Flavonoid Derivatives

**Figure 11:**
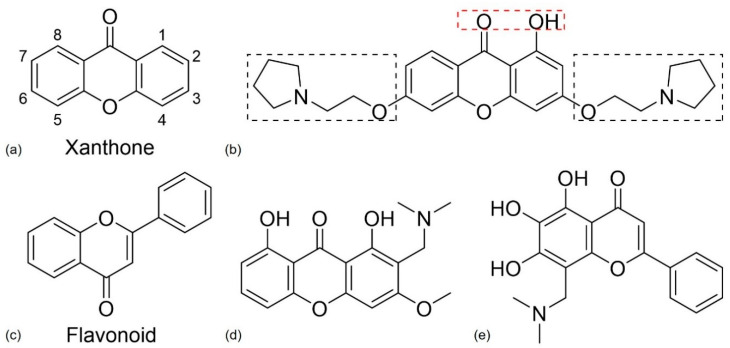
(**a**,**c**) Chemical structure of xanthone and flavonoid nucleus, respectively. (**b**) Chemical structure of the most promising xanthone derivative according to Kou et al. [111]. The substituents in the red rectangle can work as a metal chelating agent and and antioxidant, while the substituents in the black rectangle lead to antioxidant and anti-acetylcholinesterase (AChE) activity. Adapted from [111]. (**d**,**e**) Chemical structures of the most promising xanthone and flavone derivatives, respectively, obtained by Cruz et al. [101] for AD.

Xanthones, a class of oxygenated heterocyclic compounds with a dibenzo-γ-pyrone scaffold, were first isolated by Schmidt, in 1855, from the mangosteen fruit. Xanthones (Figure 11a) are commonly found in nature in higher plants, lichen, and fungi from terrestrial and marine sources [22,110]. Both natural and synthetic xanthones contain quite diverse biological activities, depending on the substitution on the scaffold and, because of that, the xanthonic scaffold is considered a “privileged structure”. Some of these compounds have shown the ability to inhibit AChE and MAO enzymes, to act as antioxidants and to inhibit the aggregation of Aβ peptides [101,110]. The majority of xanthones with multiple activities are AChE/BChE and AChE/MAO inhibitors, but there are also reports of xanthones with AChE or MAO or Aβ disaggregation and antioxidant inhibitory activities [22].

Kou et al. [111] synthesized some xanthone derivatives based on the following SAR study: a hydroxy group at C-1 and a carbonyl group on the xanthone scaffold can work as chelating agents for metal ions; the insertion of a phenolic hydroxyl and a tertiary amine moiety leads to antioxidant activity and the alkylamine side chain and the xanthone ring might interact with AChE. The chemical structure of the most promising compound of the study is presented in Figure 11b. In vitro studies confirmed its ability to act as a chelating agent and demonstrated its antioxidant activity and AChE selective inhibitory activity, closely related with the tertiary amines. This compound can react with the catalytic site and the peripheral site of AChE leading to a strong inhibitory effect [111]. These results suggest that this compound might have the potential to become a MTDL for AD treatment.

Cruz et al. [22] performed a more complete SAR study through the analysis of various xanthone derivatives with multitarget activity. The inhibition of MAO and AChE increases in the presence of a methoxy group at C-3, and for antioxidant activity a hydroxyl group on the xanthone scaffold is important. The addition of an *O*-β-glucopyranosyl group at C-8 is favorable for MAO-B inhibitory activity but prejudicial for the inhibition of MAO-A and AChE. Additionally, at C-8, the insertion of a prenyl group increases the inhibition of AChE and its hydroxylation is advantageous for antioxidant activity and inhibition of Aβ aggregation. At C-6 and C-7, a catechol group is important for AChE inhibition, antioxidative activity, and inhibition of Aβ aggregation. To obtain MTDLs with AChE, BchE, and Aβ aggregation inhibitory activity can be advantageous.

Flavonoids (Figure 11c) are polyphenolic heterocyclic compounds that can be found in plants. Flavonoids can be subdivided according to the position on the carbon of the C-ring, the degree of unsaturation and oxidation of the C-ring [112,113]. The activity of these compounds for AD targets are well known, and they demonstrate antioxidant, anti-inflammatory, neuroprotective, AChE inhibition, MAO inhibition, metal chelating, and anti-Aβ fibrillogenesis activities. These results make flavonoids a potential MTDL for AD [113].

Cruz et al. [101] synthesized xanthone and flavonoid derivatives and obtained some promising derivatives, the xanthone derivative presented in Figure 11d, and the flavone derivative presented in Figure 11e. Both compounds showed anti AChE and antioxidant activity in vitro, but further analysis is required to investigate their potential for AD therapy.

## 5. Drug Repurposing

It is extremely important to find new therapeutics for AD. However, drug development is challenging and time consuming, therefore a drug repurposing strategy seems relevant [114]. An existing drug can have more than one therapeutic effect and that is the main objective of drug repurposing, to give a new life to existing drugs. In the “new life”, a repurposed agent can act by the same mechanism of action or by a mechanism different from the traditional [114].

The interest in drug repurposing is increasing, especially in diseases with a lack of drug treatments, such as orphan diseases, and in new combination therapies [114]. A drug, to be market available, needs to have its safety and pharmacokinetic profiles well established. As a result of this, the development of an old drug for a new disease can bypass some stages of the normal drug discovery pipeline, saving money and time [115]. Repurposed drugs can be discovered by serendipity, novel insights, target searching, or clinical observation of side effects [114,115].

In current treatment for AD, an example of a repurposed drug is galantamine (Figure 3). Galantamine is an alkaloid present in *Galanthus* sp. with a complex chemical structure possessing three chiral centers. It was originally used to treat myopathies and peripheral neuropathies and for reverse neuromuscular blockade following anesthesia, because of its ability to inhibit muscle AChE and increase nerve impulse transmission. As a result of the presence of a tertiary ammonium base, it can easily penetrate the BBB and inhibit brain AChE, preventing Aβ aggregation and decreasing Aβ peptide toxicity [114,116,117].

**Figure 12:**
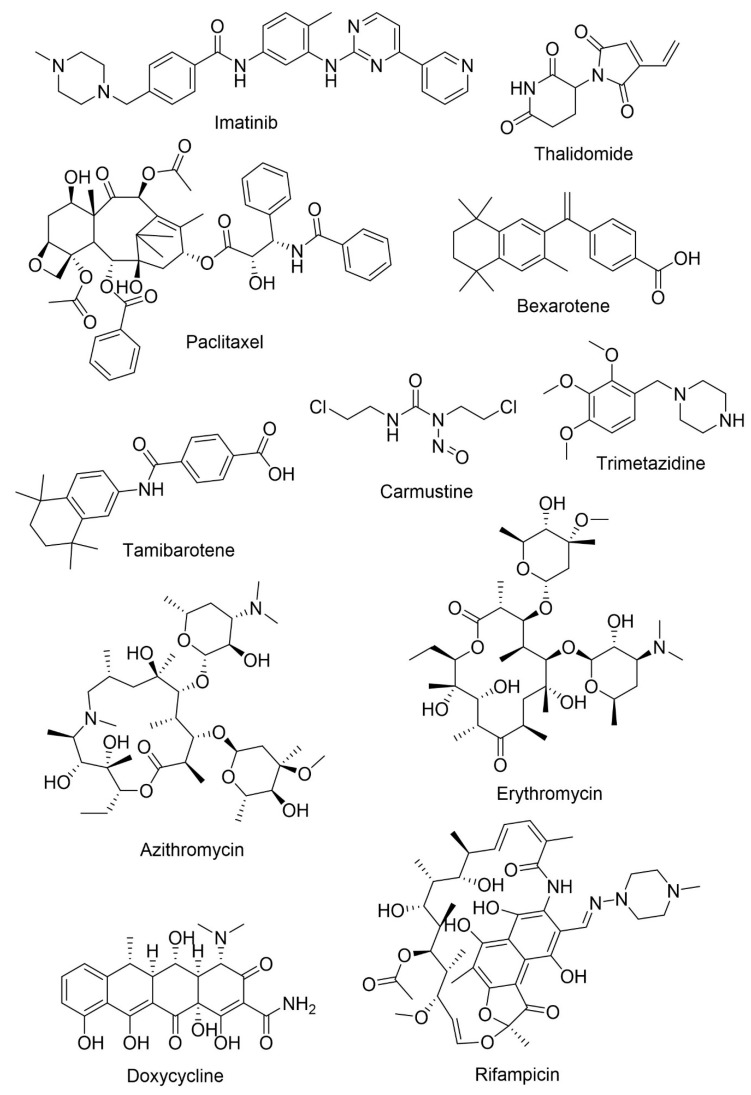

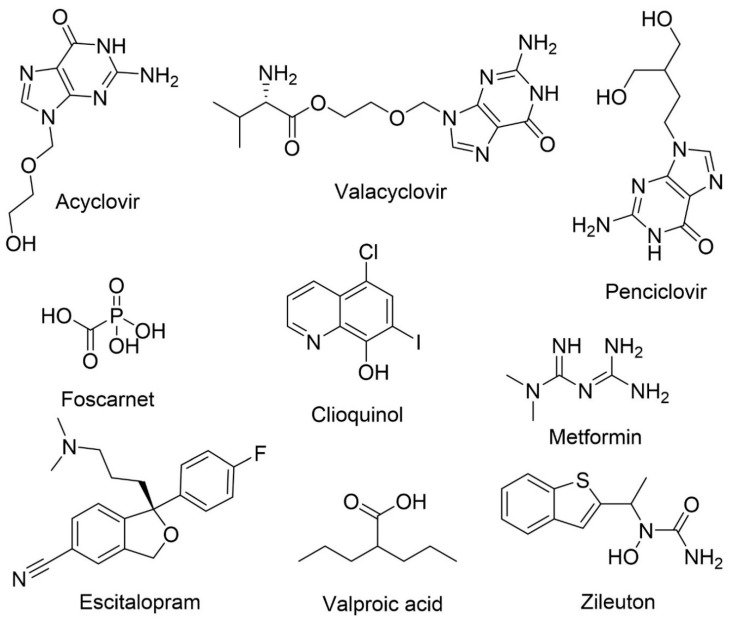
Chemical structures of potential agents for AD treatment.

Agents with the possibility of being repurposed for AD treatment are presented in Table 3 and Figure 12.

Epidemiological studies demonstrated that the risk of developing AD is lower in individuals with certain forms of cancer, like breast cancer, colorectal, and others [120]. Cancer and AD share certain common signaling pathways, such as oxidative stress, mitochondrial dysfunction, DNA damage, and compromised cell metabolism [120], and some drugs used in cancer therapy are being investigated for the treatment of AD. One example is imatinib, a selective tyrosine kinase inhibitor used in the treatment of chronic myeloid leukemia and gastrointestinal stomal tumor [118]. Imatinib acts on AD by two different mechanisms, neuroprotection and reduction of Aβ production [119]. The drug inhibits the interaction of γ-secretase activating protein blocking Aβ formation [120]. The passage of the drug through the BBB is low and it is a P-gp substrate, lowering its concentration in CNS [119]. Thalidomide, currently used in the treatment of multiple myeloma and severe erythema nodosum leprosum, possesses anti-inflammatory, neuroprotective, and anti-angiogenic activities by inhibiting cell proliferation through the inhibition of TNFα [122]. In vivo, the inhibition of TNFα by thalidomide lowers BACE1 activity reducing Aβ levels. The drug causes a decrease in glial activation and Aβ neuropathology [121].

Tau hyperphosphorylation results in reduced binding between tau and microtubules, decreasing its assembly and leading to fibrillization. Paclitaxel, a microtubule stabilizing agent approved for ovarian and breast cancer and non-small cell lung cancer, improves tau function, reducing its phosphorylation, which can be very useful for AD treatment. However, to become a drug that acts in the CNS it is necessary to increase its BBB permeability and overcome the problem associated with the efflux of the drug that is mediated by P-gp [114,123,124]. Bexarotene is a retinoid X receptor antagonist used in the treatment of cutaneous T-cell lymphoma. In vivo, bexarotene increased the concentration of APOE, reduced Aβ levels and amyloid deposition, improved cognition and reversed behavioral deficits. It is a good candidate for AD treatment, because of its good BBB permeability and safety profile [125]. Tamibarotene, also a retinoic acid receptor agonist, is a multi-target drug used in acute promyelocytic leukemia. In vivo, tamibarotene ameliorated the decrease of cortical acetylcholine, improved behavioral symptoms, such as sleep deficit and anxiety, improved memory and decreased the secretion of proinflammatory cytokines and chemokines. Compared to other retinoids, tamibarotene has fewer adverse effects [126]. Carmustine is a nitrosourea and an alkylating agent used in the treatment of malignant gliomas. It demonstrated the ability to reduce Aβ production in vivo by increasing activity in the non-amylogenic pathway and also reducing neuroinflammation, suppressing microglial activation. It can penetrate the BBB and act in CNS [120,127]. Trimetazidine is an anti-ischemic agent with antioxidant properties and is capable of inducing axonal regeneration and myelination. In vivo, trimetazidine reduced oxidative stress by increasing the activity of superoxide dismutase and catalase and decreasing malondialdehyde. The DHCR24 gene is a marker of the oxidative stress and degeneration in central neurons and it is decreased in AD patients. In the presence of trimetazidine, the expression of the DHCR24 gene is increased in the hippocampus [128].

The hypothesis of an infection being the cause in some AD cases is being studied. Among pathogens, the most common are *Herpes simplex* virus type I, *Chlamydia pneumoniae* and *Porphyromonas gingivalis*, which can cross the BBB, enter the CNS and cause neuroinflammation, acting, in this way, as a trigger or a co-factor for AD [133]. Some antimicrobials and antiviral drugs are being studied for AD treatment. Starting with antimicrobials, azithromycin and its analogue erythromycin are macrolide antibiotics that target bacterial RNA causing bacteriostasis [130]. They alter APP processing, reducing cerebral Aβ levels, and erythromycin might also have neuroprotective properties [129,130]. Tetracyclines constitute a promising multi-target therapeutic approach for AD with anti-amyloidogenic effects and neuroprotective activities [131]. Doxycycline, a second-generation tetracycline, is used in the treatment of bacterial pneumonia, syphilis, cholera, early Lyme disease, acne, and chlamydia infections. In vivo, doxycycline reduced neuroinflammation leading to memory improvement and reduced Aβ oligomers. It has been evaluated in two clinical trials. In one, the decline of cognitive abilities and functional behavior decreased, but in the second study there were no significant benefits [131]. Rifampicin is used in tuberculosis, leprosy, Legionnaires’ disease, and *Mycobacterium avium* complex. It is a neuroprotective antibiotic capable of crossing the BBB and possesses anti-inflammatory and anti-oxidative properties that modulate neuroinflammation and Aβ metabolism [132]. Rifampicin and doxycycline have already been tested together, with promising results for AD [114].

Acyclovir, used principally in the treatment of human *Herpes simplex* virus infections, and valacyclovir, its pro-drug, are two antiviral agents. Acyclovir, administered intravenously, is a nucleoside analogue that stops viral replication [133]. Penciclovir is used to treat herpesvirus infections and is a guanosine analogue. Foscarnet is also used in the treatment of herpesvirus infections and it is a DNA polymerase inhibitor [133]. Acyclovir, penciclovir and foscarnet reduced the accumulation of Aβ and phosphorylated tau protein in cell models, increasing their potential for AD treatment [114].

The metals zinc, copper, and iron are involved in the deposition and stabilization of amyloid plaques, implying that the use of chelating agents might be useful since they have the ability to dissolve amyloid deposits by preventing metal-Aβ interactions [134]. One example is clioquinol, a hydroxyquinoline with antifungal and antiprotozoal activities used as a topical formulation for the treatment of skin infections, capable of acting as a zinc, iron, and copper chelator and, by reducing the concentration of the ions, it also acts as an antioxidant [129,134]. In vivo, clioquinol caused a reduction of amyloid deposits [114].

Evidence suggests that diabetes and related features, such as insulin resistance, increase the risk of AD. As a result of this, antidiabetic treatments may be useful for AD treatment [140]. The treatment of diabetes includes several drug classes: biguanides like metformin, sulfonylureas, thiazolidinediones, sodium-glucose cotransporter 2 inhibitors, α-glucosidase inhibitors, glucagon-like peptide 1 based therapies, and insulin [140]. Metformin is a biguanide and an antihyperglycemic drug used in the treatment of type II diabetes. It reduces the concentration of blood glucose by increasing glucose uptake into the muscles and reduces gluconeogenesis. Metformin is associated with AD prevention because it prevents hyperinsulinemia, which contributes to the formation of Aβ plaques, and it also reduces inflammation and oxidative stress [135]. Mixed results were obtained with this drug in AD models. Alone, metformin reduced tau hyperphosphorylation, although, in another study, it increased the activity of β-secretase increasing Aβ levels. However, its co-administration with insulin decreased Aβ levels [129].

Citalopram, a selective serotonin reuptake inhibitor present in the clinic to treat depression, is a racemic mixture of escitalopram, the (*S*)-enantiomer which is an antidepressant, and (*R*)-citalopram, the (*R*)-enantiomer [136]. Clinical studies showed that daily administration of Citalopram was associated with a greater reduction in agitation in AD patients when compared to antipsychotic drugs, the current drugs used in therapy to treat this problem. However, the drug was associated with adverse cognitive and cardiac effects [96]. Studies demonstrated that escitalopram was responsible for serotonin reuptake inhibition and non-clinical antidepressant activity while (*R*)-citalopram was associated with cognitive worsening, and with a lower probability to reduce agitation. Escitalopram is therefore, being studied as a potential treatment of agitation in AD patients [136].

Valproic acid, a histone deacetylase, is an antiepileptic drug used in the treatment of seizures and bipolar disorder acting as a multitarget agent in AD being anti-inflammatory and neuroprotective [137]. Mice treated with valproic acid showed improvement in memory, less accumulation of Aβ deposits, and a decrease in inflammation. This possibly implies that valproic acid can modulate microglial activity via inhibition of proinflammatory cytokine production and induce apoptosis of microglial cells, reducing inflammation [137,138].

The enzyme 5-lipoxygenase, which catalyzes two steps in the biosynthesis of leukotrienes, is upregulated in AD patients and, in vivo, modulated Aβ levels and tau metabolism. Zileuton, a specific inhibitor of 5-lipoxygenase, is used to treat asthma but was shown to reduce Aβ, tau phosphorylation, and delay the onset of cognition impairments in mouse models of AD [139].

Some of the drugs identified above are already in clinical studies for AD treatment. This strategy is proving beneficial in the identification of new drugs and provides hope for a new treatment for this complicated disease.

## 6. Conclusions and Future Perspectives

The most promising strategies for drug discovery for AD treatment were highlighted in this review. Among the diverse possibilities, natural products remain a very interesting source of new molecules, and particular emphasis was given to MNPs, displaying new and unique chemical structures and demonstrating useful biological activities with potential for AD treatment. Another approach considered drug repurposing, requiring less time and monetary investment, which are important due to the urgency in finding an effective treatment for AD. Nevertheless, drug repurposing faces some major challenges that need to be overcome, which include intellectual property rights, older drug indications, and other technical challenges. Combination therapies and MTDL are two strategies that aim to modulate more than one target, although in different ways. With AD being a multifactorial disease, these two strategies are important approaches in this field.

Every new study or clinical trial increases our knowledge about AD, meaning that the gap between what we currently know and a possible cure becomes smaller. Early this year, a sign of hope emerged with the discovery of a new molecule (GV-971), showing a new mechanism of action. This compound completed phase III clinical trials and is awaiting approval to enter the market.

**Figure 13:**
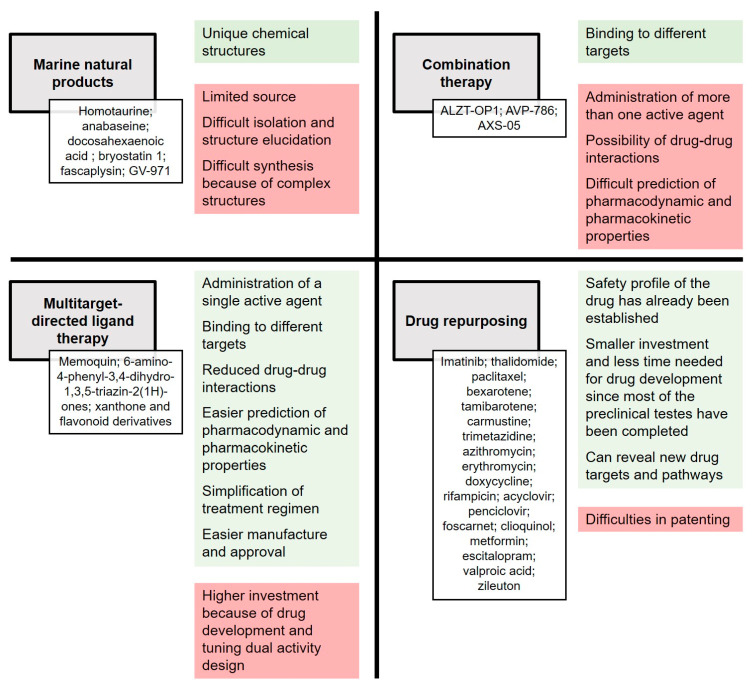
Advantages, in green, and disadvantages, in red, of the four drug discovery strategies addressed; marine natural products (MNP), combination therapy, multitargeted-directed ligand therapy (MTDL) and drug repurposing. In white are the mentioned agents with potential to become a drug candidate for AD treatment.

The outlined approaches have several advantages and disadvantages, summarized in Figure 13. Considering all the analyzed compounds and strategies, we propose that the best approach to follow will be the design of dual BACE-1 and GSK-3β inhibitors, as they will be able to target two of the central enzymes in AD pathology. Finally, we consider that future research in the field should focus not only on identifying effective drugs, but also on improving the early diagnosis of AD so that treatment can commence early in order to halt the development of this progressive neurodegenerative disease.

## Figures and Tables

**Table 1:** List of the main properties of marine natural products and derivatives in the pipeline for AD treatment.

Chemical Structure	Chemical Family	Relevant Numbers	Therapeutic Purpose	Pharmacokinetic Profile	In the Pipeline	Reference
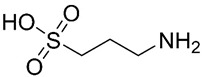 Homotaurine	Amino sulfonate	NCT0031491	Anti-amyloid	Safe Oral administration causes gastrointestinal irritation Crosses the blood-brain barrier (BBB)	Failed phase III clinical trials	[33,35,36]
	Particularities: It binds to Aβ peptide through the sulfonated head, preventing the progression of amyloid cascade and maintaining Aβ in a stable conformation. In vitro, its ability to inhibit Aβ aggregation and fibrillogenesis, decrease the levels of Aβ_40_ and Aβ_42_ and interfere with toxic oligomers formation was demonstrated. In clinical trials, it reduced Aβ_42_ levels in AD patients following three months of treatment, demonstrated benefits in cognitive function and reduction of hippocampus volume loss. The benefit in cognitive function was more prominent in APOE4+ patients.					
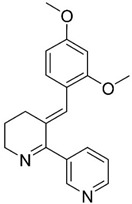 GTS-21	Benzylidene	NCT00414622	Agonist activity against nicotinic acetylcholine receptors (nAChRs)	Lipophilic compound with a high permeation through the BBB. Hydroxy-metabolites of the compound have poorer BBB penetration	Phase II clinical trials	[37,38,39,40]
	Particularities: It is an anabaseine derivative more potent and selective for α7 nAChRs than the MNP anabaseine. The three rings lie in different planes, unlike the two rings of anabaseine. It possesses some stimulant effect following oral administeration. Effect may be caused by the metabolites. In vitro, GTS-21 reduced Aβ through the suppression of γ-secretase activity and promotion of microglial Aβ phagocytosis. In vivo, neuroprotective effects from amyloid toxicity, apoptosis and necrosis, and improvement in cognitive functions were observed.					
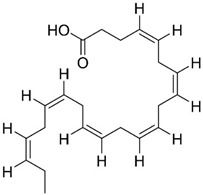 DHA	Omega-3 fatty acid	NCT00440050 NCT03613844	Reducing Aβ formation Improve synaptic function	Low bioavailability and suffers oxidation	Phase III clinical trials	[41,42,43,44,45,46,47,48,49,50,51]
	Particularities: Docosahexaenoic acid (DHA) is essential for brain health and neurodevelopment and its reduction is associated with AD. In vivo, DHA supplementation reduced tau hyperphosphorylation, neurotoxic damage associated with Aβ plaques, and Aβ deposition through the shifting of APP metabolism via the non-amyloidogenic pathway. The effects are dependent on the stage of AD progression and positive effects were verified in patients in the initial stage of AD. Neuroprotectin D1 (NPD1) is a bioactive DHA-derivative that showed anti-inflammatory, anti-amyloidogenic, and anti-apoptotic activities, also useful for the treatment of the initial stage of AD.					
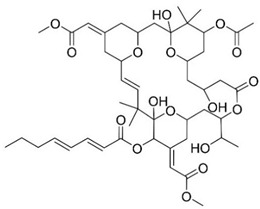 Bryostatin 1	Macrolide lactone	NCT03560245	PKC modulator Neuroprotection	Safe and well tolerated	Phase II clinical trials	[52,53,54,55,56,57]
	Particularities: Bryostatin-1 activates PKC isozyme epsilon (PKCε), causing its downregulation. Consequently, degradation of Aβ, activation of α-secretase generating the synaptogenic non-toxic soluble amyloid-β protein precursor α, reduction of GSK3-β activity that leads to decreasing hyperphosphorylation of tau ensues. Bryostatin-1 elevates synaptic growth factors in the brain possible causing neuroprotective effects. Structure-activity relationship (SAR) studies demonstrated that the 20-membered macrolactone ring is essential, but the elimination of the A-ring and B-ring exocyclic olefin is possible. The C-26 free hydroxyl and C-1 carbonyl group are necessary for interaction and affinity, respectively. The C-19 hydroxyl group might interact with the lipid bilayer and C-3 hydroxyl group is important for the molecules conformation. The C-9 region can be modified to alter pharmacokinetic characteristics and C-20 can be used to form analogues. A phase IIa safety and tolerability test showed that bryostatin-1 increases PKCε and is safe, with a favorable pharmacokinetic and an initial cognitive improvement with a single dose.					
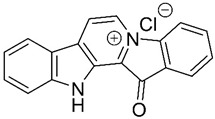 Fascaplysin	Bis-indole alkaloid	-	AChE inhibition P-glycoprotein (P-gp) induction	Possibility in crossing BBB	-	[58,59,60,61]
	Particularities: The principal interaction of fascaplysin with AChE is a π-π interaction. It binds parallelly in AChE active site gorge, with B-ring orientating to the catalytic site and D-ring orientating towards peripheral site. The quaternary status of the C-ring nitrogen is essential for P-gp activity. Fascaplysin increases P-gp activity leading to a higher Aβ clearance. In vitro, 9-methylfascaplysin is more potent than fascaplysin in inhibiting Aβ fibrillation and protection against the neurotoxicity associated with Aβ oligomer. It acts as an antioxidant, can prevent cognitive dysfunction, decreases neuroinflammation and reduces tau hyperphosphorylation.					
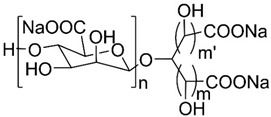 GV-971 *n* = 1–9; *m* = 0,1 or 2; *m*’ = 0 or 1	Acidic oligosaccharide	NCT02293915	Anti-inflammatory	Crosses BBB via Glut-1 transporter Low oral bioavailability	Completed successfully phase III clinical trial	[62,63,64,65]
	Particularities: Restoration of the normal microbial profile leading to a reduction of neuroinflammation associated with T helper type 1 (Th1) cells. Studies, in vivo, demonstrated its ability to reduce Aβ deposition, tau phosphorylation, improve cognitive function and, reduce Th1 cells, thereby alleviating neuroinflammation. In clinical trials, increased Aβ clearance and improved cognitive function were observed.					

**Table 2:** Combination therapies in phase III clinical trials for AD treatment [23].

Agent	Characteristics	Mechanism of Action	AD Target	Particularities	Relevant Numbers	References
ALZT-OP1	Cromolyn + ibuprofen	Mast cell stabilizer and anti-inflammatory activity	Amyloid and inflammation	The co-administration of cromolyn and ibuprofen was safe and well tolerated; ibuprofen acts on γ-secretase modulation, instead of inhibiting cyclooxygenase; cromolyn acts on microglia, increasing Aβ phagocytosis, in vivo, and decreases the aggregation of Aβ peptide, in vitro.	NCT02547818	[85,86,87,88,89,90]
AVP-786	Deuterated (d6)-dextromethorphan + quinidine	Activation of sigma-1 receptors, NDMA receptor antagonist and cytochrome P450 2D6 inhibition	Agitation	Quinidine decreases dextromethorphan metabolization; deuterium also decreases dextromethorphan metabolization, reducing the necessary dose of quinidine.	NCT02442765 NCT02442778 NCT02446132	[91,92]
AXS-05	Bupropion + dextromethorphan	NMDA receptor antagonist, sigma-1 receptor agonist, a serotonin and noradrenaline reuptake inhibitor, and cytochrome P450 2D6 inhibition	Agitation	Safe and well tolerated; bupropion and dextromethorphan possess synergist effects; increases the amount of dopamine, serotonin glutamate and noradrenaline; increases plasma levels of dextromethorphan.	NCT03226522	[93,94,95]

**Table 3:** Candidates for drug repurposing in AD treatment.

Agent	Current Treatment	Mechanism in AD	Particularities	References
Imatinib	Chronic myeloid leukemia and gastrointestinal stomal tumor	Neuroprotection and reduction of Aβ formation	Inhibits the interaction of γ-secretase activating protein with γ-secretase	[118,119,120]
			Low BBB permeability and suffers efflux mediated by P-gp	
Thalidomide	Multiple myeloma and severe erythema nodosum leprosum	Anti-inflammatory, neuroprotection, and anti-angiogenic activities	Decreases glial activation and Aβ neuropathology through the inhibition of tumor necrosis factor-α (TNFα)	[121,122]
			Poor BBB permeability	
Paclitaxel	Ovarian and breast cancer and non-small cell lung cancer	Antimitotic agent	Reduces tau phosphorylation	[114,123,124]
			Poor BBB permeability and P-gp-mediated efflux	
Bexarotene	Cutaneous T-cell lymphomas	Anti-amyloid	Increases APOE concentration, reduces Aβ levels and amyloid deposition and improves cognition	[125]
			High BBB permeability	
Tamibarotene	Acute promyelocytic leukemia	Immunomodulatory activity	Improves cortical acetylcholine decrease, decreases proinflammatory cytokines and chemokines, improves behavioral symptoms	[126]
			Good BBB permeability	
Carmustine	Brain cancer	Anti-amyloid	Reduces Aβ production and neuroinflammation	[120,127]
			Its lipophilic structure confers good BBB permeability	
Trimetazidine	Angina pectoris	Neuroprotection	Increases the expression of DHCR24 and reduces oxidative stress	[128]
			Crosses the BBB	
Azithromycin	Bacterial infections	Anti-amyloid	Alters APP processing leading to a reduction in Aβ levels	[129,130]
Erythromycin	Bacterial infections	Anti-amyloid	Alters APP processing leading to a reduction in Aβ levels	[129,130]
			Possible neuroprotective effect	
Doxycycline	Bacterial pneumonia, syphilis, cholera, early Lyme disease, acne, and chlamydia infections	Anti-amyloid	Reduces neuroinflammation and reduces Aβ oligomers	[131]
			Crosses BBB and has a safe clinical profile	
Rifampicin	Tuberculosis, leprosy, Legionnaires’ disease and *Mycobacterium avium* complex	Anti-amyloid	Modulates neuroinflammation and Aβ metabolism	[132]
			Crosses the BBB	
Acyclovir	Human herpes virus infections	Anti-amyloid and anti-tau	Reduces Aβ accumulation and phosphorylated tau protein in cell models	[114,133]
			The prodrug, valacyclovir, is hydrolyzed in vivo to acyclovir which has the ability to cross BBB	
Penciclovir	Human herpes virus infections	Anti-amyloid and anti-tau	Reduces Aβ accumulation and phosphorylated tau protein in cell models	[114,133]
Foscarnet	Human herpes virus infections	Anti-amyloid and anti-tau	Reduces Aβ accumulation and phosphorylated tau protein in cell models	[114,133]
Clioquinol	Skin infections	Anti-amyloid	Reduces amyloid deposits in vivo by preventing metal-Aβ interactions	[114,129,134]
			Acts as a zinc, iron and copper chelator and, by reducing the concentration of the ions, it also acts as an antioxidant	
Metformin	Antihyperglycemic drug	Anti-amyloid	Prevents hyperinsulinemia, reduces inflammation and oxidative stress	[129,135]
			Observation of mixed results. In vivo, metformin reduced tau hyperphosphorylation but there is evidence that it can increase BACE-1 activity increasing Aβ levels. The co-administration with insulin decreased Aβ levels	
Escitalopram	Antidepressant	Agitation	Escitalopram is the (*S*)-enantiomer of citalopram	[96,136]
			Currently in phase III clinical trials for agitation reduction	
Valproic acid	Antiepileptic	Anti-inflammatory and neuroprotection	In vivo, improves memory, reduces the accumulation of Aβ deposits and decreases inflammation.	[137,138]
			Possible modulation of microglia	
Zileuton	Antiasthma	Anti-amyloid and anti-tau	Specific inhibitor of 5-lipoxygenase	[139]
			Reduces β-amyloid and tau phosphorylation, and improves cognitive function	
